# Evaluation value of ultrasound on gastrointestinal function in patients with acute heart failure

**DOI:** 10.3389/fcvm.2024.1475920

**Published:** 2024-11-25

**Authors:** Ruyi Hao, Ye Zheng, Qing Zhao, Jie Chen, Ruiqi Fan, Peng Chen, Na Yin, Huai Qin

**Affiliations:** ^1^ Department of Diagnostic Ultrasound, Beijing Anzhen Hospital, Capital Medical University, Beijing, China; ^2^ Department of Emergency, Beijing Anzhen Hospital, Capital Medical University, Beijing, China

**Keywords:** acute heart failure, ultrasound, gastrointestinal function, gastrointestinal wall thickness, gastrointestinal symptom

## Abstract

**Objective:**

To study the changes in gastrointestinal wall thickness, blood flow, motility, and symptoms in patients with acute heart failure, and to assess gastrointestinal function by ultrasound.

**Methods:**

In this study, patients diagnosed with acute heart failure were selected as the study group, and healthy individuals were selected as the control group. Both groups collected general data and completed the Chinese version of the gastrointestinal symptom rating scale. Ultrasonography was used to measure several abdominal vascular and gastrointestinal-related indicators. Statistical analysis used grouped comparison and correlation analysis.

**Results:**

The study group scored higher than the control group in total score, lower abdominal symptom score, constipation score, and difficult defecation score (*Z* = −2.828, −2.022, −2.015, −2.015, all *P* < 0.05). The hepatic vein diameter, superior mesenteric vein inner diameter and wall thickness of the ascending colon in the study group were significantly higher than those in the control group (*t* = 9.543, *P* < 0.001; *t* = 2.277, *P* = 0.025; *Z* = −2.062, *P* = 0.039). Antral contraction amplitude, antral contraction frequency, motility index, jejunal peristalsis frequency, and ascending colon peristalsis frequency were significantly lower in the study group compared to the control group (*Z* = −2.571, −4.196, −3.681, −5.451, −4.061, all *P* < 0.001). The wall thickness of the antrum, jejunum, and ascending colon were positively correlated with the diameter of the hepatic vein (*r* = 0.394, *P* = 0.011; *r* = 0.352, *P* = 0.024; *r* = 0.450, *P* = 0.003). Motility index and ascending colon peristalsis frequency were positively correlated with the peak velocity of superior mesenteric vein (*r* = 0.456, *P* = 0.029; *r* = 0.507, *P* = 0.007). The wall thickness of the jejunum was positively correlated with the peak velocity of superior mesenteric artery (*r* = 0.330, *P* = 0.035). Peak velocity of superior mesenteric artery, antral contraction frequency, and jejunal peristalsis frequency were negatively correlated with the reflux score (*r* = −0.409, *P* = 0.038; *r* = −0.423, *P* = 0.032; *r* = −0.409, *P* = 0.038). The wall thickness of the ascending colon was positively correlated with the reflux score (*r* = 0.414, *P* = 0.035).

**Conclusion:**

This study found that patients with acute heart failure exhibited thickening of the gastrointestinal wall and generally reduced gastrointestinal motility, with predominantly lower abdominal symptoms. These findings indicate that ultrasound can effectively monitor the gastrointestinal structure and function of patients with acute heart failure, which is expected to provide help for clinical diagnosis and treatment.

## 1 Introduction

Acute heart failure (AHF) is a major health problem affecting millions of people worldwide. In recent years, efforts have been made to find more effective strategies to prevent and change the course of the disease, but the results are still unsatisfactory (1). AHF is a complex clinical syndrome involving complications across multiple systems, with gastrointestinal complications being particularly under- discussed and poorly understood.

Gastrointestinal-related phenomena are prevalent in patients with AHF and are closely related to higher morbidity and mortality (2). The correlation between AHF and the gastrointestinal system is increasingly recognized by cardiologists. A better understanding of this relationship will aid in guiding existing management and treatment.

At present, the indicators for evaluating gastrointestinal function are scattered, and there is no unified and standardized evaluation index. Assessment mainly relies on clinical symptoms, laboratory evaluation indicators (such as kinetic detection indicators, gastrointestinal hormones, and specific biomarkers), and other evaluation indicators (including molecular probes, nuclear magnetic resonance imaging, pathology, and isotope labeling) (3). Clinical symptoms lack objectivity, laboratory evaluation indicators are scattered and no specific marker accurately reflects gastrointestinal function. Additionally, other evaluation indicators are expensive, complicated to operate, and some are invasive and radioactive. Therefore, there is currently no reliable method to evaluate gastrointestinal function in patients with AHF to meet clinical needs.

Ultrasound is a convenient, inexpensive, and non-invasive method for evaluating both normal and pathological gastrointestinal tracts. It offers high temporal and spatial resolution, can be performed bedside, and provides real-time information on gastrointestinal motility, blood flow, edema, filling, and emptying (4). In addition, the Chinese version of the Gastrointestinal Symptom Rating Scale (GSRS) is a classic questionnaire for patients with gastrointestinal diseases. It demonstrates high measurement performance, good reliability and validity, and responsiveness, making it suitable for assessing gastrointestinal symptoms in the general population and evaluating therapeutic effects (5).

The main purpose of this study is to study the changes in gastrointestinal wall thickness, blood flow, motility, and symptoms in patients with AHF, aiming to assess the value of ultrasound in evaluating gastrointestinal function in these patients.

## 2 Methods

### 2.1 Research object

We sequentially enrolled patients presenting with AHF at the emergency department of our hospital, starting from November 2023, up to reaching a total of *n* = 50 patients. This was achieved by August 2024. Among the 50 screened patients, 5 patients were excluded due to previous gastrointestinal lesions or gastrointestinal surgery, and 4 patients were excluded due to severe abdominal distension. According to the study protocol, we planned to collect the same number of healthy controls as the study group as a control group. This study was approved by the hospital ethics committee and obtained the informed consent of all subjects.

### 2.2 Inclusion and exclusion criteria

Inclusion criteria for the study group: patients diagnosed with AHF in the emergency department (including AHF and acute exacerbation of chronic heart failure) who were conscious and able to cooperate with the scale survey, and able to take oral fluids. Inclusion criteria for the control group: healthy individuals who had been ruled out of hypertension, heart disease and without obvious gastrointestinal symptoms were selected as the control group. Exclusion criteria for the study group and the control group: patients with known gastrointestinal diseases or suspected gastrointestinal lesions found during gastrointestinal ultrasonography; patients under 18 years old or pregnant; overweight appearance or severe abdominal distension, leading to poor imaging; and patients with incomplete clinical data.

### 2.3 Ultrasound examination

The ultrasound scanner used in this study (GE Logiqe, GE Healthcare, USA) is equipped with two ultrasound transducers: a curvilinear 1.4–5.7 MHz transducer (C1-5) and a linear 3.4–12.6 MHz transducer (L4-12t).

**Figure 1:**
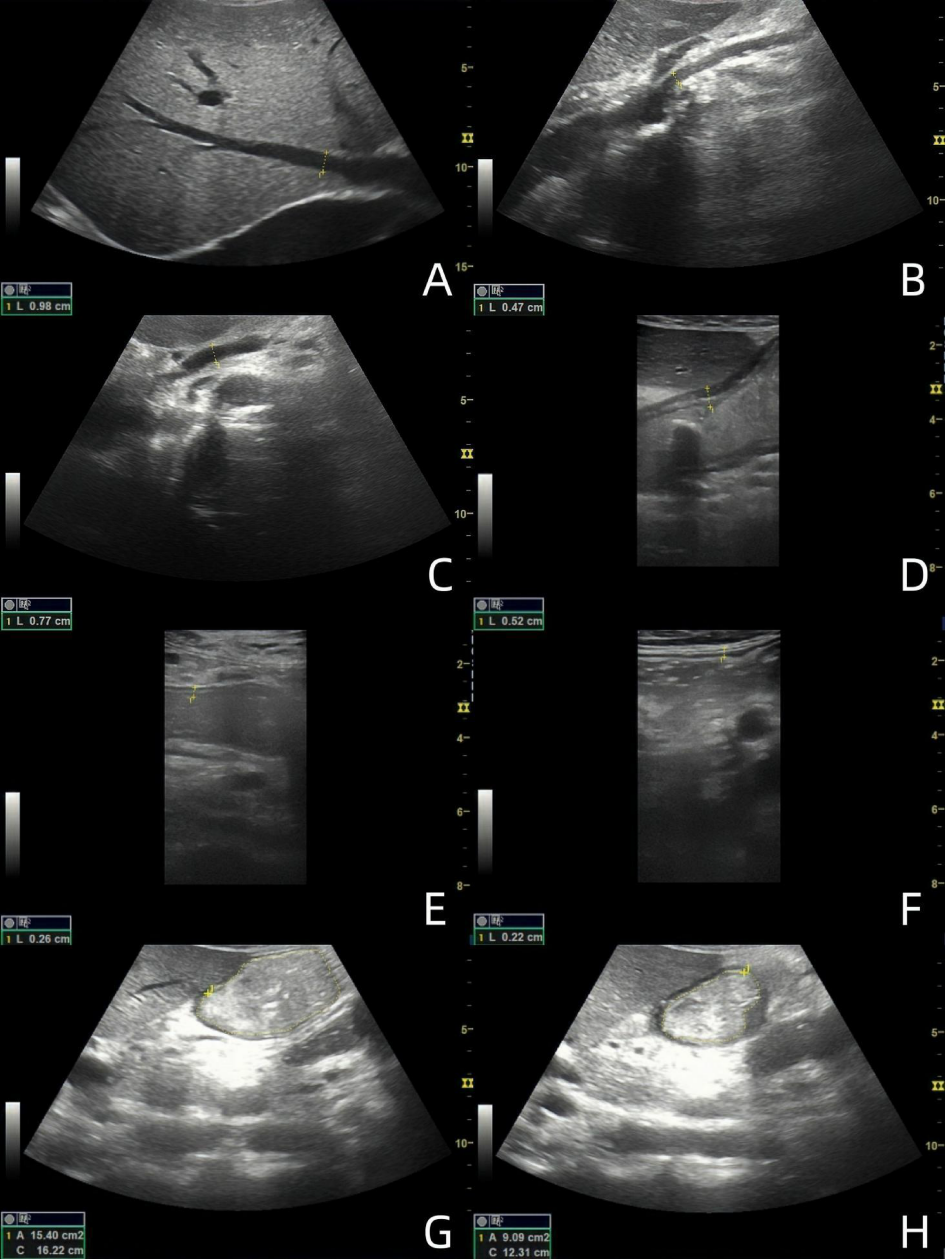
Ultrasound images of 49-year-old male patients with acute heart failure. **(A)** Maximum diameter of hepatic vein; **(B)** SMA inner diameter; **(C)** SMV inner diameter; **(D)** antral wall thickness; **(E)** Jejunal wall thickness; **(F)** Ascending colon wall thickness; **(G)** Maximal diastolic area of gastric antrum; **(H)** Minimal contraction area of gastric antrum. (The images are sorted from left to right and from top to bottom as **A-H**).

All subjects fasted for more than 6 h. The convex array probe was used to scan the abdomen of the subjects in both groups. First, three hepatic veins (HVs) were displayed, the image was appropriately enlarged, and the inner diameters of the three HVs were measured, with the maximum value recorded (Figure 1A). Subsequently, the superior mesenteric artery (SMA) and superior mesenteric vein (SMV) were displayed, and the image was enlarged appropriately to observe the lumen structure. The inner diameter was measured within 1 cm from the starting position (Figures 1B,C). Color Doppler imaging was performed to observe the blood flow filling, and spectrum Doppler imaging was performed to measure the peak velocity corresponding to the inner diameter measurement site.

The subjects were instructed to drink 300 ml of warm water while in a semi-recumbent position. The convex array probe was placed under the xiphoid process, and the probe marker oriented towards the subject's head to show the sagittal plane of the midline of the abdomen. The probe was slightly lateralized, and the gastric antrum, abdominal aorta, and SMA were displayed at the same time to evaluate the contraction of the gastric antrum (Figures 1G,H). The probe was stabilized 3 min to observe and calculate the contraction frequency per minute of the gastric antrum and to measure the antral contraction amplitude (ACA). ACA = (the maximum antral diastolic area - the minimum antral systolic area)/the maximum antral diastolic area. The motility index (MI) was then calculated as MI = Antral contraction frequency (ACF) (The number of antral contractions per minute) × ACA × 2 (6). The convex array probe was then moved to the umbilical level to display the long axis of the jejunum. The probe was observed for 2 min to calculate the peristaltic frequency of the jejunum per minute. The peristaltic frequency of jejunum = the number of jejunum peristalsis in 2 min ÷ 2. Subsequently, the convex array probe was moved to the right abdomen to display the ascending colon. The probe was stabilized for 2 min to calculate the peristaltic frequency of the ascending colon per minute. The peristaltic frequency of ascending colon = the number of ascending colon peristalsis in 2 min ÷ 2. The high-frequency linear array probe was then placed at the corresponding body surface positions of the gastric antrum, jejunum, and ascending colon, respectively. The images of the regions of interest were enlarged, and the wall thickness was measured from the hypoechoic layer of the muscularis propria to the hypoechoic layer of the mucosa. Measurements were taken as vertically as possible, avoiding colonic haustra and mucosal folds in the colon. Each measurement was repeated three times, and the average value was calculated (Figures 1D–F).

### 2.4 The Chinese version of gastrointestinal symptom rating scale (GSRS)

The Chinese version of GSRS was used to evaluate the gastrointestinal symptoms of the two groups of patients (Table 1). The questionnaire was completed by 26 patients in the study group and 26 patients in the control group. It consists of 15 questions, including abdominal pain, heartburn, acid reflux, abdominal hunger pain, nausea and vomiting, bowel sounds, bloating, hiccups (belching), increased flatulence, decreased defecation frequency, increased defecation frequency, loose stool, stool induration, urgency of defecation, and incomplete defecation. These symptoms were divided into eight dimensions: abdominal pain, upper abdominal symptoms, lower abdominal symptoms, reflux, dyspepsia, diarrhea, constipation, and difficulty in defecation. Each question is scored from 1 (totally no symptoms) to 7 (especially serious), and each dimension score and total score were recorded.

**Table 1:** Chinese version of the gastrointestinal symptom rating scale (GSRS).

Have the following symptoms in the past week	Score						
	Totally not	A little bit	A small amount	Middle degree	More obvious discomfort	Relatively severe	Specially serious
Abdominal pain	1	2	3	4	5	6	7
Heartburn	1	2	3	4	5	6	7
Acid reflux	1	2	3	4	5	6	7
Abdominal hunger and pain	1	2	3	4	5	6	7
Nausea and vomiting	1	2	3	4	5	6	7
Bowel sounds	1	2	3	4	5	6	7
Bloating	1	2	3	4	5	6	7
Hiccups (belching)	1	2	3	4	5	6	7
Increased exhaust	1	2	3	4	5	6	7
Decreased defecation frequency	1	2	3	4	5	6	7
Increased defecation frequency	1	2	3	4	5	6	7
Loose stool	1	2	3	4	5	6	7
Stool induration	1	2	3	4	5	6	7
Urgency of defecation	1	2	3	4	5	6	7
Incomplete defecation	1	2	3	4	5	6	7

### 2.5 Observation indicators

(1) General information: including the age, gender, height, weight, body mass index (BMI) of the two groups of subjects and the B-type natriuretic peptide (BNP), cardiac ejection fraction (EF), tricuspid annulus systolic displacement (TAPSE) within 3 days of ultrasound examination in the AHF group; (2) HV inner diameter, SMA and SMV inner diameter and peak velocity; (3)ACF, ACA, MI, wall thickness of the antrum; (4) Jejunal peristalsis frequency, ascending colon peristalsis frequency, the wall thickness of the jejunum and the thickness of the ascending colon wall; (5) Chinese version of GSRS total score and eight dimensions score.

### 2.6 Statistical analysis

SPSS 22.0 software was used for statistical analysis. Measurement data conforming to normal distribution was expressed as mean ± standard deviation (±SD), and the independent sample *t*-test was used for comparison between groups. Measurement data not conforming to normal distribution was expressed as [M(QL, QU)], and the rank sum tests was used for comparison between groups. The χ^2^ tests was used to compare the count data, and Spearman correlation analysis was used for correlation analysis. *P*-value of <0.05 was considered statistically significant. GPower was used for a *post hoc* power calculation.

## 3 Results

### 3.1 Baseline characteristics comparison

The study group included 41 patients with heart failure, while 41 healthy individuals were selected as the control group. The general clinical characteristics of patients, including age, gender, height, weight, and BMI, were retrospectively collected and compared between the groups. The results presented in Table 2 show that there are no statistically significant differences in these clinical characteristics between the AHF and control groups.

**Table 2:** Comparison of basic data between AHF group and healthy physical examination group (*n* = 82).

Basic information/groups	Acute heart failure group (*n* = 41)	Health examination group (*n* = 41)	χ^2^/*t*	*P*
Age (years)	65.39 ± 13.28	62.05 ± 9.94	1.290	0.201
Gender (male/female)	24/17	23/18	0.050	0.823
Height (cm)	164.67 ± 8.18	164.24 ± 6.70	0.249	0.804
Weight (kg)	67.07 ± 14.19	66.05 ± 9.63	0.352	0.726
BMI (kg/m^2^)	25.03 ± 5.37	24.28 ± 2.91	0.743	0.461
BNP (pg/mL)	1,994 (964, 3,467)			
EF (%)	37 (22, 55)			
TAPSE (mm)	17 (15, 18.5)			

### 3.2 The Chinese version of GSRS score comparison

Regarding the Chinese version of the GSRS score, the AHF group had higher total scores, lower abdominal symptom scores, constipation scores, and difficult defecation scores compared to the healthy physical examination group (*Z* = −2.828, −2.022, −2.015, −2.015, all *P* < 0.05) (Table 3).

**Table 3:** Comparison of the Chinese version of GSRS score between the AHF group and healthy physical examination group (*n* = 52).

Score/groups	AHF group (*n* = 26)	Health examination group (*n* = 26)	*Z*	*P*
Total score	22.5 (17, 31)	19 (17, 20)	−2.828	0.005*
Abdominal pain score	3 (3, 3.5)	3 (3, 4)	−0.920	0.358
Upper abdominal symptom score	7 (7, 10.5)	7 (7, 8)	−0.051	0.959
Lower abdominal symptom score	8.5 (6, 14)	7 (6, 8)	−2.022	0.043*
Dyspepsia score	5 (4, 10)	4.5 (4, 6)	−1.375	0.169
Reflux score	2 (2, 4.5)	2 (2, 3)	−0.213	0.831
Constipation score	7 (3, 8)	4 (3, 5)	−2.015	0.044*
Defecation difficulty score	6 (2, 7)	3 (2, 4)	−2.015	0.044*
Diarrhea score	3 (3, 3)	3 (3, 3)	−0.033	0.974

* Means *P* < 0.05.

### 3.3 Comparison of ultrasound parameter

Regarding ultrasound parameters, there were significant differences between the AHF group and the healthy examination group. Specifically, the HV diameter, SMV inner diameter and ascending colon wall thickness of the AHF group were significantly higher than those of the healthy physical examination group (*t* = 9.534, *P* < 0.001; *t* = 2.277, *P* = 0.025; *Z* = −2.062, *P* = 0.039). Additionally, the AHF group had significantly lower values for ACA, ACF, MI, jejunal peristalsis frequency, and ascending colon peristalsis frequency compared to the healthy examination group (*Z* = −2.571, −4.196, −3.681, −5.451, −4.061, *P* < 0.001) (Table 4).

**Table 4:** Comparison of ultrasonic parameters between AHF group and healthy physical examination group (*n* = 82).

Ultrasonic parameters/Groups	AHF group (*n* = 41)	Health examination group (*n* = 41)	*Z*/*t*	*P*
HV inner diameter (cm)	1.06 ± 0.28	0.59 ± 0.14	9.534	<0.001*
SMA inner diameter (cm)	0.67 ± 0.12	0.63 ± 0.13	1.598	0.114
Peak velocity of SMA (cm/s)	145.07 ± 42.62	157 (122, 179)	0.839	0.401
SMV inner diameter (cm)	0.86 ± 0.20	0.75 ± 0.22	2.277	0.025*
Peak velocity of SMV (cm/s)	37 (32, 53)	39.85 ± 11.96	−0.483	0.629
Wall thickness of the antrum (cm)	0.38 ± 0.13	0.40 ± 0.08	−0.673	0.503
Antral contraction amplitude (%)	44.25 (10.50, 54.05)	61.70 (38.20, 67.50)	−2.571	<0.001*
Antral contraction frequency (times/min)	2 (1, 3)	3 (3, 4)	−4.196	<0.001*
Motility index	1.67 (0.17, 3.15)	3.53 ± 1.78	−3.681	<0.001*
Wall thickness of the jejunum (cm)	0.13 (0.11, 0.21)	0.15 ± 0.05	−0.502	0.615
Jejunal peristalsis frequency (times/min)	1 (0, 4)	6.27 ± 2.43	−5.451	<0.001*
Ascending colon wall thickness (cm)	0.17 (0.14, 0.25)	0.15 ± 0.03	−2.062	0.039*
Ascending colon peristalsis frequency (times/min)	0 (0, 2)	2 (2, 3)	−4.061	<0.001*

* Means *P* < 0.05.

### 3.4 Correlation analysis between ultrasound parameters in patients with AHF

**Figure 2:**
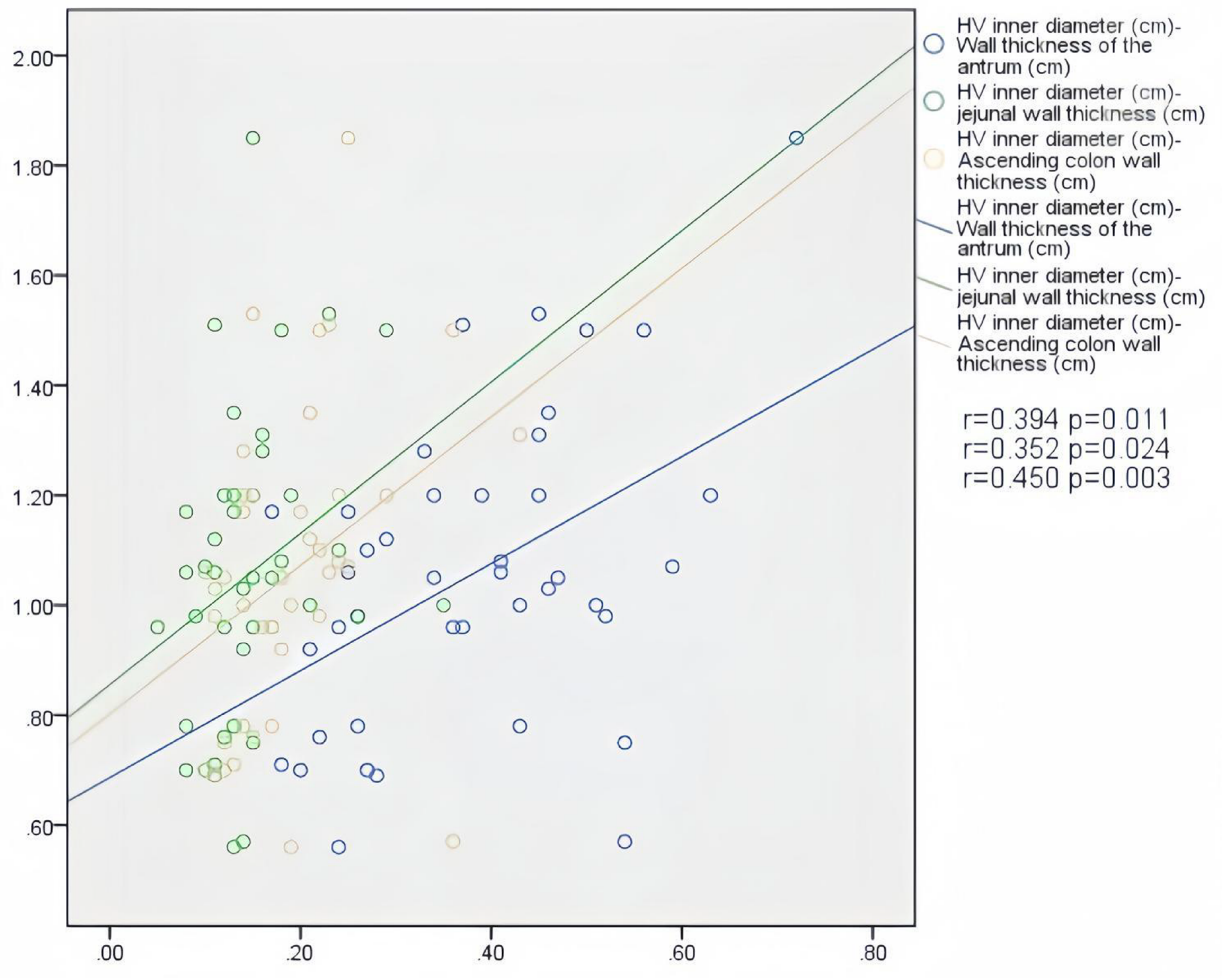
Correlation between HV diameter and gastrointestinal wall thickness in patients with acute heart failure.

**Figure 3:**
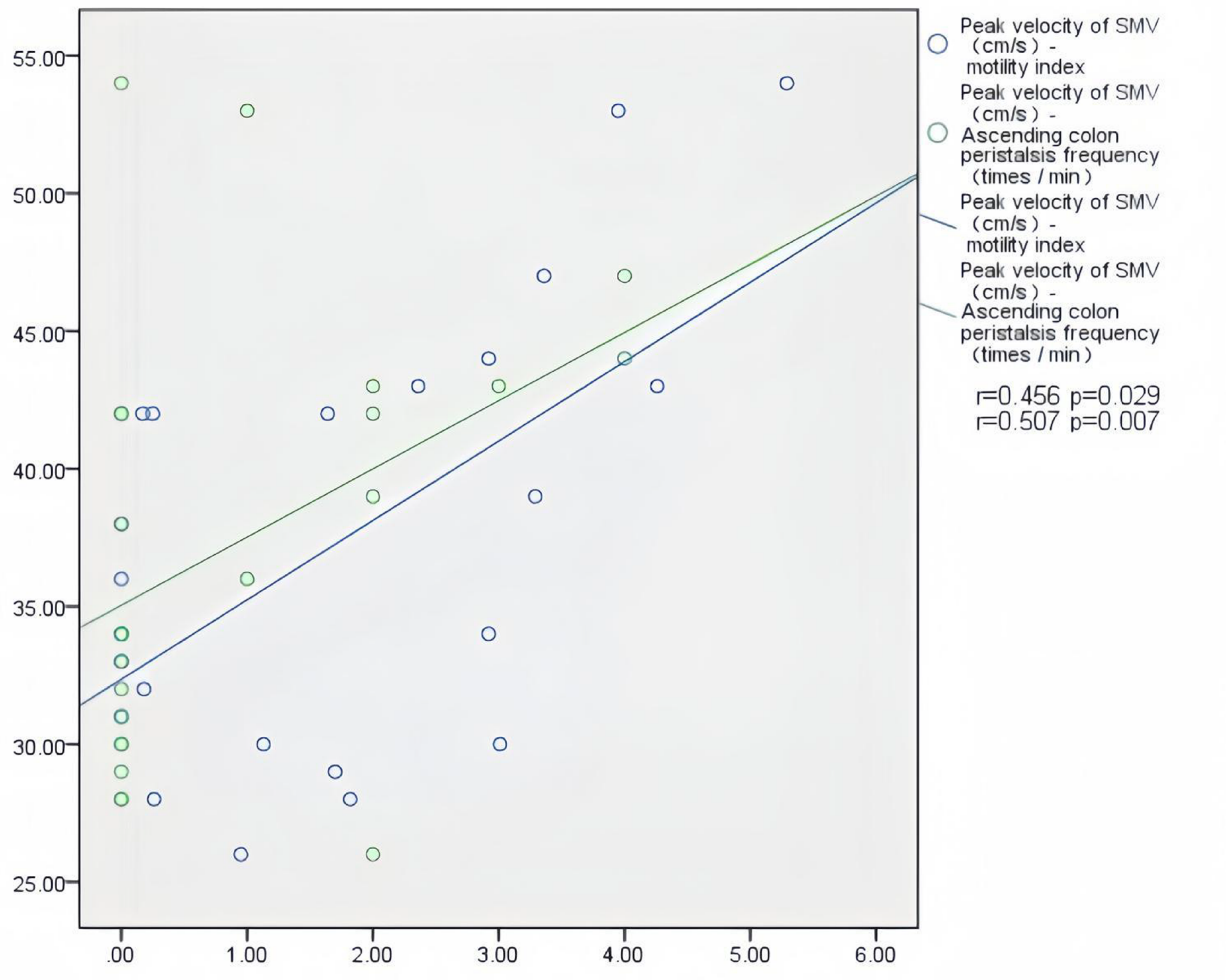
Correlation between motility index, ascending colon peristalsis frequency and SMV peak velocity in patients with acute heart failure.

**Figure 4:**
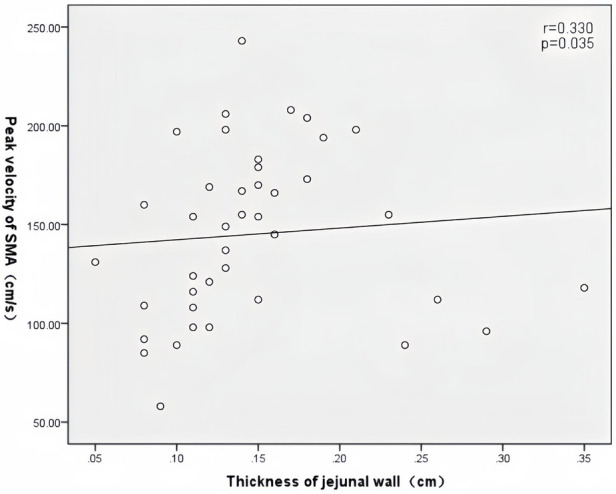
Correlation between jejunal wall thickness and SMA peak velocity in patients with acute heart failure.

In patients with AHF, the wall thickness of the antrum, jejunum, and ascending colon were positively correlated with the inner diameter of HV (*r* = 0.394, *P* = 0.011; *r* = 0.352, *P* = 0.024; *r* = 0.450, *P* = 0.003) (Figure 2); motility index and ascending colon peristalsis frequency were positively correlated with SMV peak velocity (*r* = 0.456, *P* = 0.029; *r* = 0.507, *P* = 0.007) (Figure 3), and the wall thickness of the jejunum was positively correlated with the peak velocity of SMA (*r* = 0.330, *P* = 0.035) (Figure 4) (Table 5).

**Table 5:** Correlation analysis of ultrasound parameters in patients with AHF.

Ultrasonic parameters		HV inner diameter	SMA inner diameter	Peak velocity of SMA	SMV inner diameter	Peak velocity of SMV
Wall thickness of the antrum	*r*	0.394	−0.093	0.127	0.046	−0.052
	*P*	0.011*	0.564	0.429	0.775	0.746
Antral contraction amplitude	*r*	−0.104	0.339	0.258	0.331	0.315
	*P*	0.577	0.062	0.161	0.069	0.084
Antral contraction frequency	*r*	−0.214	0.291	0.288	0.076	0.274
	*P*	0.284	0.140	0.145	0.705	0.166
Motility index	*r*	−0.217	0.338	0.342	0.329	0.456
	*P*	0.321	0.115	0.110	0.125	0.029*
Jejunal wall thickness	*r*	0.352	−0.139	0.330	−0.009	0.126
	*P*	0.024*	0.388	0.035*	0.953	0.432
Jejunal peristalsis frequency	*r*	−0.308	0.053	0.088	0.078	0.071
	*P*	0.119	0.794	0.663	0.700	0.726
Ascending colon wall thickness	*r*	0.450	−0.142	0.078	0.252	−0.227
	*P*	*0*.003*	0.374	0.626	0.112	0.154
Ascending colon peristalsis frequency	*r*	−0.103	−0.258	0.134	−0.069	0.507
	*P*	0.609	0.195	0.507	0.732	0.007*

* Means *P* < 0.05.

### 3.5 Correlation analysis between ultrasound parameters and the Chinese version of GSRS score in patients with AHF

**Figure 5:**
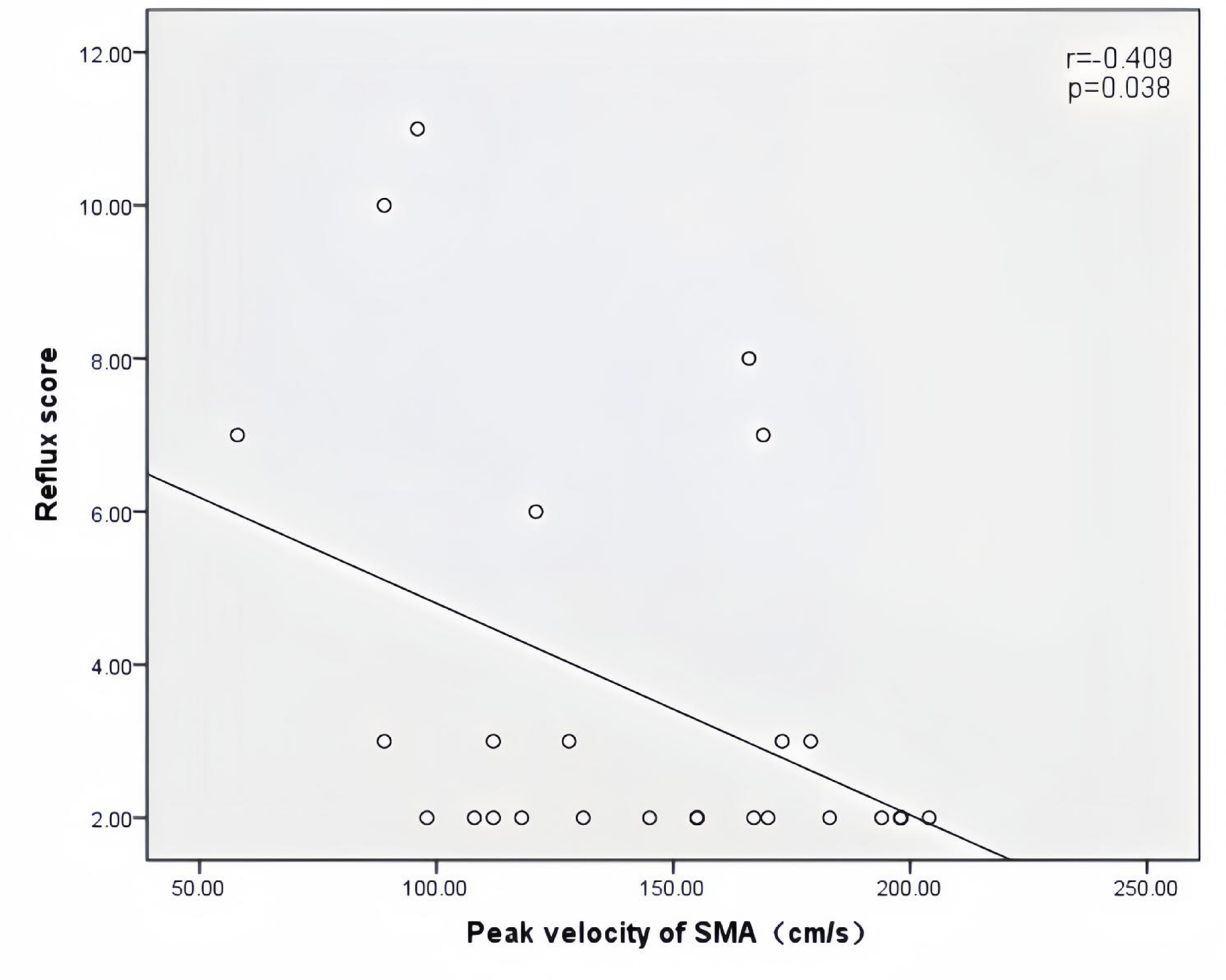
Correlation between peak velocity of SMA and the reflux score in patients with acute heart failure.

**Figure 6:**
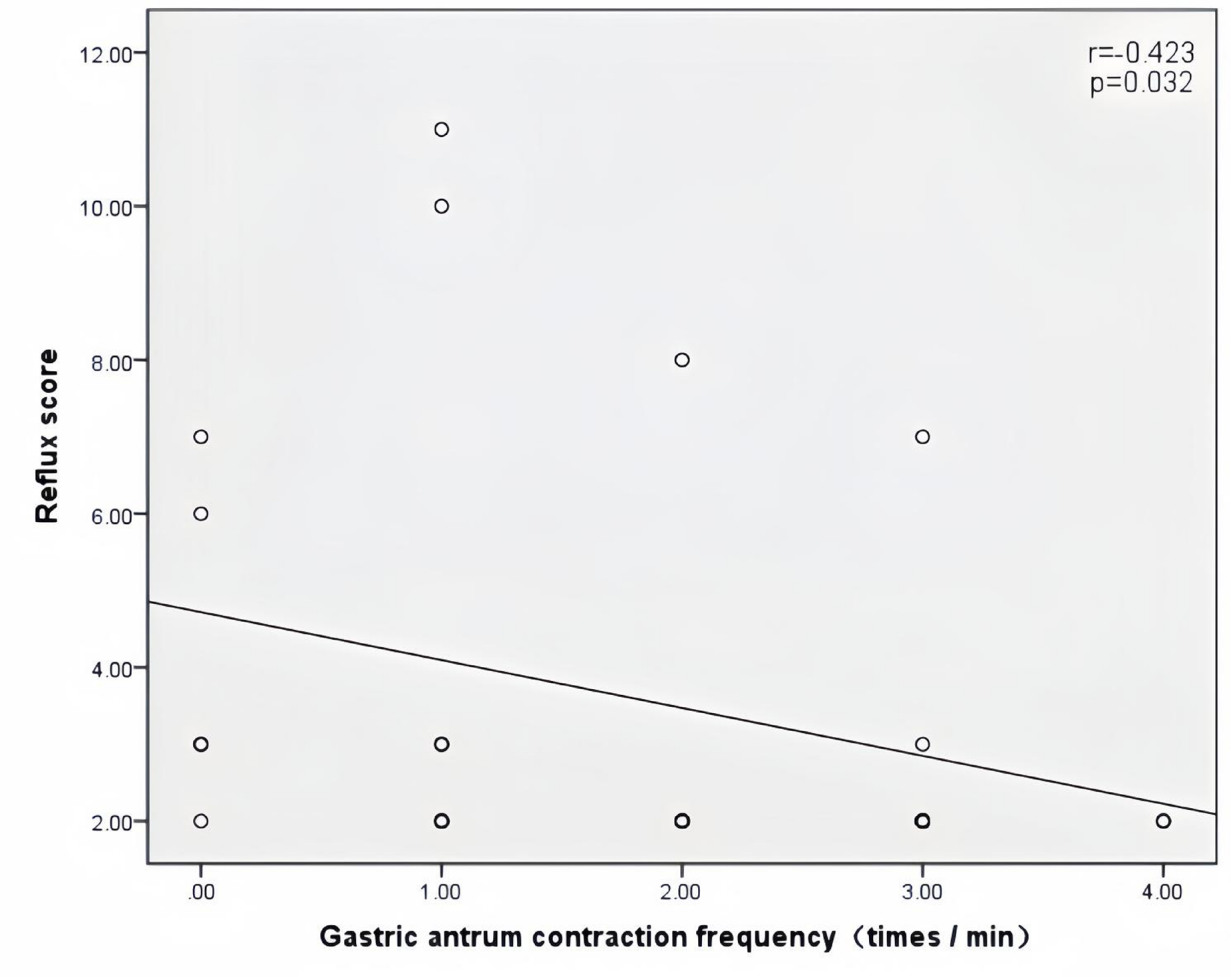
Correlation between the antral contraction frequency and the reflux score in patients with acute heart failure.

**Figure 7:**
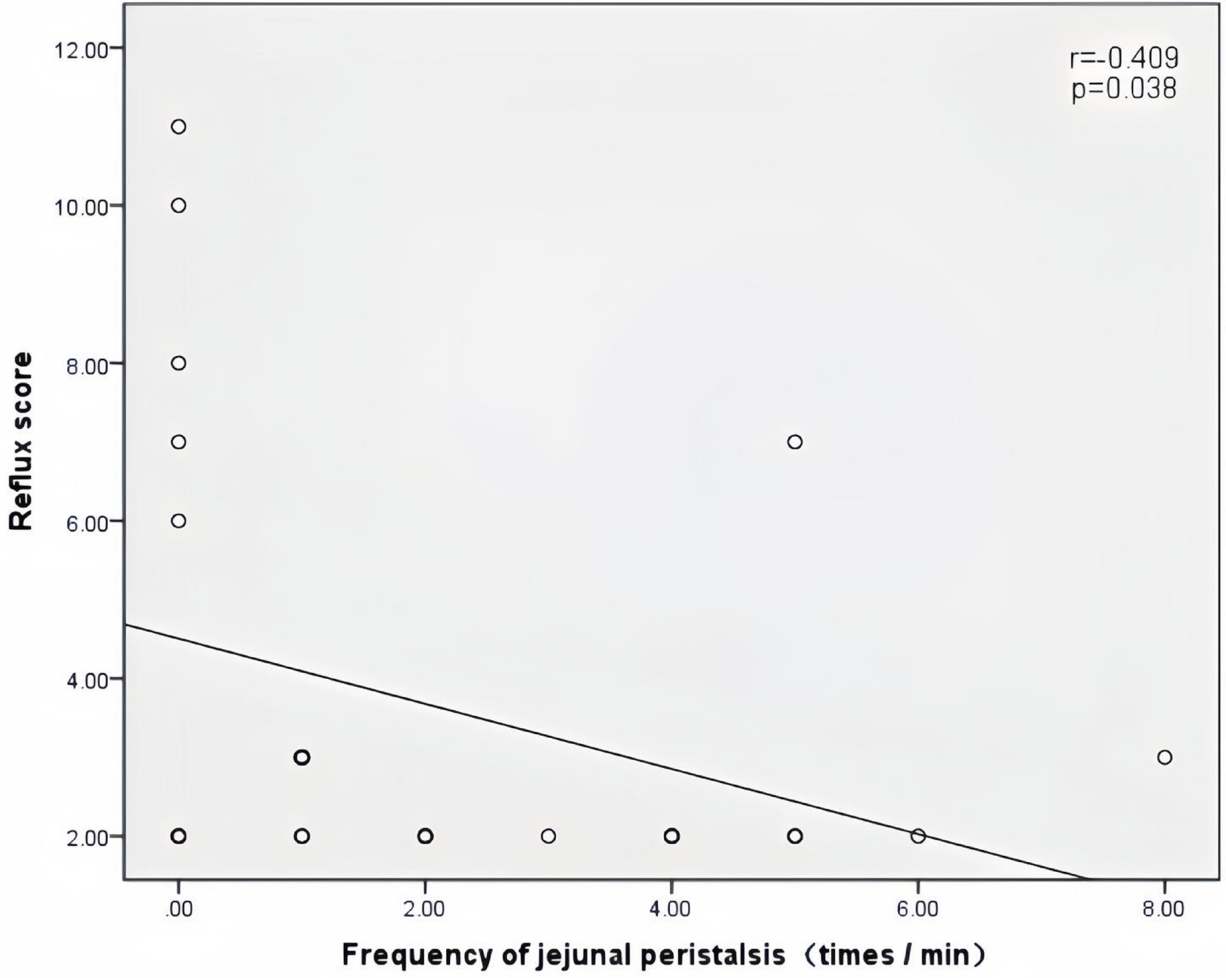
Correlation between the jejunal peristalsis frequency and the reflux score in patients with acute heart failure.

**Figure 8:**
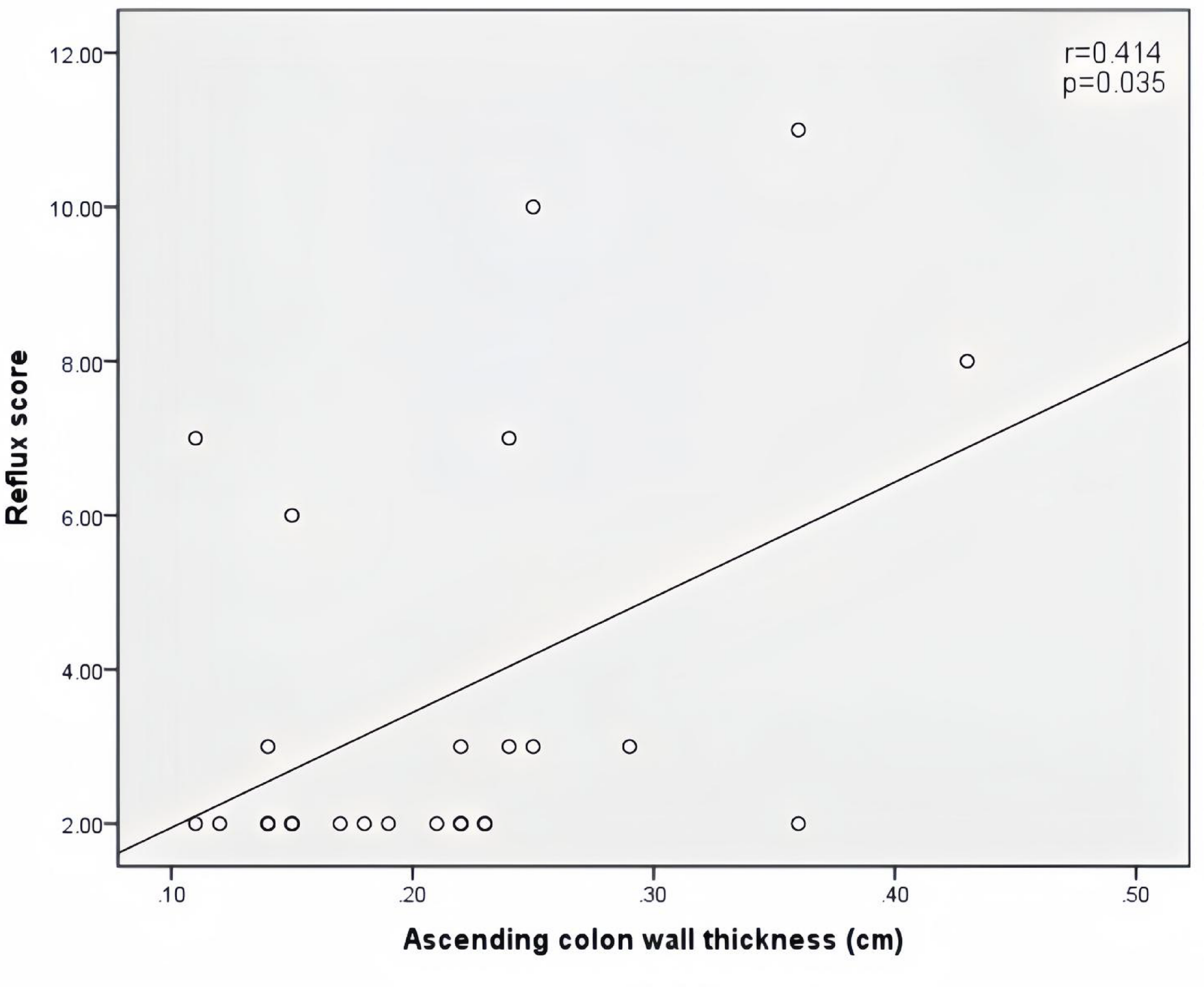
Correlation between ascending colon wall thickness and the reflux score in patients with acute heart failure.

Peak velocity of SMA, ACF, and jejunal peristalsis frequency were negatively correlated with the reflux score (*r* = −0.409, *P* = 0.038; *r* = −0.423, *P* = 0.032; *r* = −0.409, *P* = 0.038) (Figures 5–7). Conversely, the wall thickness of the ascending colon was positively correlated with the reflux score (*r* = 0.414, *P* = 0.035) (Figure 8) (Table 6).

**Table 6:** Correlation analysis between ultrasound parameters and the Chinese version of GSRS score in patients with AHF.

Ultrasonic parameters	Score	Total score	Abdominal pain score	Upper abdominal symptom score	Lower abdominal symptom score	Dyspepsia score	Reflux score	Constipation score	Defecation difficulty score	Diarrhea score
HV inner diameter	*r*	−0.004	0.138	−0.136	0.025	−0.111	0.166	−0.028	−0.028	−0.031
	*P*	0.986	0.501	0.509	0.904	0.588	0.418	0.892	0.892	0.879
SMA inner diameter	*r*	−0.179	0.047	0.180	−0.164	0.072	−0.335	−0.139	−0.139	0.052
	*P*	0.383	0.820	0.379	0.424	0.726	0.094	0.498	0.498	0.799
Peak velocity of SMA	*r*	−0.273	−0.073	−0.073	−0.228	−0.029	−0.409	−0.107	−0.107	−0.080
	*P*	0.177	0.725	0.722	0.263	0.888	0.038*	0.603	0.603	0.699
SMV inner diameter	*r*	−0.084	0.065	−0.005	−0.104	−0.044	0.087	0.251	0.251	0.023
	*P*	0.682	0.753	0.980	0.614	0.830	0.671	0.216	0.216	0.912
Peak velocity of SMV	*r*	−0.186	−0.082	−0.022	−0.117	−0.248	−0.138	−0.221	−0.221	0.305
	*P*	0.364	0.692	0.917	0.570	0.222	0.502	0.278	0.278	0.129
Wall thickness of the antrum	*r*	0.018	0.310	−0.036	−0.011	−0.015	0.038	0.067	0.067	−0.118
	*P*	0.930	0.123	0.861	0.956	0.941	0.854	0.746	0.746	0.567
Antralal contraction amplitude	*r*	−0.226	−0.056	−0.251	−0.313	−0.149	−0.397	0.156	0.156	−0.138
	*P*	0.312	0.803	0.261	0.156	0.507	0.067	0.488	0.488	0.540
Antral contraction frequency	*r*	0.030	0.160	0.002	−0.134	−0.021	−0.423	0.240	0.240	−0.139
	*P*	0.884	0.434	0.991	0.514	0.919	0.032*	0.237	0.237	0.500
Motility index	*r*	−0.181	−0.044	−0.158	−0.273	−0.077	−0.422	0.181	0.181	−0.142
	*P*	0.420	0.847	0.482	0.218	0.732	0.050	0.421	0.421	0.529
Wall thickness of the jejunum	*r*	−0.263	−0.057	0.091	−0.112	−0.050	−0.144	−0.321	−0.321	0.183
	*P*	0.194	0.782	0.660	0.587	0.807	0.483	0.110	0.110	0.371
Jejunal peristalsis frequency	*r*	−0.182	−0.362	−0.170	−0.298	0.077	−0.409	0.235	0.235	−0.238
	*P*	0.374	0.069	0.407	0.139	0.709	0.038*	0.248	0.248	0.241
Ascending colon wall thickness	*r*	0.305	0.310	−0.007	0.130	−0.194	0.414	0.347	0.347	0.020
	*P*	0.129	0.123	0.971	0.527	0.342	0.035*	0.083	0.083	0.921
Ascending colon peristalsis frequency	*r*	0.098	0.244	−0.161	0.086	0.039	0.024	0.196	0.196	−0.123
	*P*	0.635	0.229	0.431	0.676	0.851	0.908	0.338	0.338	0.551

* Means *P* < 0.05.

### 3.6 Correlation analysis between ultrasound parameters, GSRS score, and BNP, EF, TAPSE in patients with AHF

There was no significant correlation between ultrasound parameters, GSRS Score, and BNP, EF, or TAPSE in patients with AHF (Table 7).

**Table 7:** Correlation analysis between ultrasonic parameters, GSRS score, and BNP, EF, and TAPSE in patients with AHF.

Ultrasonic parameters	BNP		EF		TAPSE	
	*r*	*p*	*r*	*p*	*r*	*p*
HV inner diameter	−0.014	0.934	0.213	0.188	−0.226	0.160
SMA inner diameter	−0.022	0.895	−0.079	0.626	0.244	0.129
Peak velocity of SMA	−0.098	0.549	0.121	0.458	0.238	0.139
SMV inner diameter	−0.051	0.752	0.109	0.503	0.168	0.299
Peak velocity of SMV	−0.133	0.414	0.053	0.747	0.174	0.282
Wall thickness of gastric antrum	0.071	0.665	−0.009	0.954	−0.247	0.124
Antral contraction amplitude	0.237	0.198	0.055	0.768	0.058	0.757
Antral contraction frequency	0.085	0.674	0.084	0.678	0.207	0.300
Motility index	0.156	0.489	0.066	0.772	0.018	0.938
Wall thickness of the jejunum	0.103	0.525	−0.135	0.405	−0.119	0.464
Jejunal peristalsis frequency	0.209	0.296	−0.100	0.620	0.129	0.520
Ascending colon wall thickness	0.039	0.811	−0.088	0.590	−0.311	0.050
Ascending colon peristalsis frequency	−0.044	0.828	−0.120	0.552	−0.045	0.822
Total score	−0.205	0.316	−0.140	0.494	−0.252	0.215
Abdominal pain score	−0.078	0.707	−0.173	0.398	−0.132	0.520
Upper abdominal symptom score	−0.127	0.535	−0.160	0.434	−0.026	0.899
Lower abdominal symptom score	−0.185	0.365	−0.134	0.514	−0.178	0.385
Dyspepsia score	−0.075	0.717	−0.043	0.837	−0.030	0.885
Reflux score	−0.329	0.100	−0.110	0.593	−0.194	0.342
Constipation score	−0.085	0.679	0.051	0.806	−0.196	0.338
Defecation difficulty score	−0.085	0.679	0.051	0.806	−0.196	0.338
Diarrhea score	−0.097	0.638	−0.012	0.955	−0.023	0.911

### 3.7 *Post hoc* power calculation

The sample size of both the study group and the control group in this study is 41. With *α* = 0.05, and assuming an effect size of 0.5 (effect size = 0.2 means small; effect size = 0.5 means medium; effect size = 0.8 means large), the obtained statistical power is 0.61.

## 4 Discussion

Heart failure is defined by medical history and clinical signs. NT-proBNP is now recognized as an important supportive factor for diagnosis and monitoring treatment response. Echocardiography can help identify specific causes or rule out complications (such as left ventricular thrombus) or classify HF into HFrEF and HFpEF. In this study, the combination of patients' medical history and clinical signs with a significant increase in BNP levels and a significant decrease in EF values was used to diagnose AHF.

In patients with AHF, the total score of GSRS, as well as scores for lower abdominal symptoms, constipation, and difficulty defecation were significantly higher compared to those in the healthy examination group. In this study, patients with acute heart failure exhibited thicker ascending colon walls, and those with greater intestinal wall thickness showed a significant reduction in ascending colon peristalsis. This thickness was associated with more pronounced symptoms of constipation and difficulty in defecation, and some cases even led to intestinal obstruction. These issues may be attributed to edema of the intestinal wall, increased thickness of intestinal tissue caused by collagen deposition, and the intestinal injury caused by chronic perfusion deficiency (7). Such factors likely exacerbate intestinal barrier dysfunction and dynamic disorders, manifesting as the corresponding lower abdominal symptoms, constipation, and difficult defecation.

Regarding the thickness of gastrointestinal wall, this study found that only the wall thickness of the ascending colon was significantly different between the two groups. This may be due to the fact that the colon is supplied by the SMA and the inferior mesenteric artery and their branches, making it heavily reliant on the collateral vascular system. Any interruption of forward blood flow in AHF may damage this fragile system, leading to hypoperfusion, ischemic injury, and ultimately intestinal necrosis. In addition, the colon is more susceptible to hypotension and low intestinal flow directly caused by AHF, because compared to other parts of the gastrointestinal tract, the colon has a poorer capacity for autoregulation. Other parts of the gastrointestinal tract have more effective compensatory mechanisms to maintain adequate perfusion (8–10). Inadequate perfusion of the colon destroys the intestinal barrier, increasing epithelial permeability, which can lead to bacterial translocation, especially of anaerobic microbes. This results in systemic inflammation and edema, causing thickening of the colon wall (2). Our previous research demonstrated that the difference in gastrointestinal wall between the heart failure group and the healthy control group was statistically significant (11). In this study, only the wall thickness of the ascending colon showed a significant difference between the two groups, whereas the wall thicknesses of the gastric antrum and the jejunum did not reach statistical significance, which may be caused by sample bias.

In both the study group and the control group, we did not find any obvious stenosis of SMA, or obvious obstruction of HV and SMV. The inner diameter of HV in patients with AHF was significantly larger than that in the healthy group, indicating that secondary liver damage is common in patients with AHF. A prospective study showed that, compared with the control group, the blood flow in the SMA, IMA, and celiac trunk was significantly reduced in the heart failure group, with systolic blood flow being reduced by 58%, 55%, and 57%, respectively (12). Other studies have shown that intestinal blood flow is reduced in patients with heart failure, which correlates with the severity of heart failure. In particular, the blood flow in the celiac trunk, SMA, and inferior mesenteric artery in patients with cachexia was significantly reduced (13). Reduced cardiac output and adaptive redistribution of systemic blood flow during heart failure may lead to intestinal hypoperfusion and ischemia (14, 15). The microstructure of the intestinal villi, which forms a plexiform structure, is ideal for optimizing nutrient absorption. However, it is also vulnerable to the shunt of oxygenated blood through the bottom of the villi, leaving the tips relatively ischemic (16). Consequently, mucosal acidosis can be observed in approximately half of patients with decompensated heart failure (17). In addition, ischemia-reperfusion injury plays a critical role when blood supply is disrupted. Ischemia-reperfusion injury can diminish intestinal barrier function, increase intestinal permeability, and promote bacterial translocation. Furthermore, intestinal ischemia-reperfusion injury can lead to the production of cytokines that may damage systemic tissues (18). Incomplete visceral cell resuscitation is associated with the development of multiple organ failure and increased mortality in critically ill patients (19). Although there was no significant difference in SMA and SMV-related indexes between the two groups in this study, the mean peak velocity of SMA and SMV in the AHF group was lower than that in the control group. This could be due to the inclusion of patients with relatively low-severity AHF in this study, resulting in less pronounced changes in gastrointestinal structure. Additionally, the small sample size of this study renders this study explorative and dictates the need for further, appropriately powered studies.

ACA, ACF, MI, jejunal peristalsis frequency, and ascending colon peristalsis frequency were significantly lower in the AHF group compared to the control group. These findings indicate that the patients with AHF experience reduced gastrointestinal motility and dysfunction.

The intestinal epithelium serves as a selective barrier that permits the absorption of nutrients, electrolytes, and water while preventing the entry of toxins, antigens, and microorganisms from the intestinal lumen into the systemic circulation. Intestinal barrier function is maintained by a balanced intestinal flora, a intact mucosa, and a competent immune system. Disruption of one or more of these protective mechanisms can lead to bacterial translocation, where bacteria or bacterial metabolites, such as endotoxin, cross the intestinal mucosa and spread to mesenteric lymph nodes or further organs like liver and spleen (14). Bacterial endotoxins and metabolites may enter the systemic circulation, triggering an immune response and inflammation (14, 20, 21), which contributes to the onset and progression of HF (22, 23). In addition, endotoxin and inflammatory cytokines can increase intestinal permeability (24–27), creating a vicious cycle of intestinal microbial endotoxin translocation, systemic inflammation and worsening heart failure. Early studies on heart failure have observed mucosal barrier dysfunction (28) and increased permeability of the small and large intestines. It has been suggested that the vicious cycle of intestinal dysfunction leads to myocardial and microcirculation dysfunction, potentially leading to further intestinal dysfunction (29, 30). In addition, we found that 4 out of the 41 patients with AHF in this study developed intestinal obstruction, representing nearly 10% of the study group. This outcome is attributed to multiple factors, including impaired intestinal motility and barrier function, inflammation, dysbacteriosis, and alterations in the intestinal microbiota (31). At present, the treatment methods for intestinal obstruction mainly include fasting, rehydration, anti-infective therapy, continuous gastrointestinal decompression, and enema. However, in severe cases, some patients may experience symptoms such as shock and dehydration, necessitating further interventions like laparotomy. Postoperative care involves nutritional support and early mobilization of patients to promote the recovery of gastrointestinal function (32). Surgery prolongs the hospitalization time, potentially increases related complications, and greatly affects the quality of life. Therefore, it is crucial to promptly assess the gastrointestinal function of patients with heart failure and monitor its dynamics in real-time. Therefore, ultrasound can fulfill this requirement and provides significant guidance for clinical treatment.

As one of the main branches of the hepatic portal vein, SMV mainly collects the blood of the right colon and flows back into the right liver. HV then gathers blood from the liver parenchyma and finally drains into the inferior vena cava. In cases of right heart dysfunction, liver congestion ensues due to impaired hepatic venous return, leading to HV dilation and increased portal vein pressure. This results in a widened portal vein system and reduced blood flow velocity. Therefore, in AHF, HV dilation can cause liver congestion, manifesting as abdominal pain, nausea, and vomiting, with accelerated gastric contractions exacerbating gastrointestinal symptoms. Further dilation of SMV, leads to gastrointestinal congestion and thickening of the gastrointestinal wall, contributing to gastrointestinal symptoms and clinical signs such as bowel sounds, abdominal distension, and indigestion. Multiple studies have also shown that intestinal swelling in patients with heart failure contributes to these gastrointestinal symptoms (12). Additionally, another study has shown that gastrointestinal symptoms in heart failure patients may be caused by intestinal motility disorders secondary to ischemia (12).

This study also supports this view. Patients have ischemic edema of the intestinal wall, which can cause several changes in measured parameters, including a decreased peak velocity of the SMA and SMV. At the same time, the patient's intestinal peristalsis slows down, and lower abdominal symptoms increase. It can be seen that the structure, blood flow, function, and symptoms of the gastrointestinal tract in AHF are closely related and influence each other. However, no significant correlation was found between ultrasound parameters, BNP values and cardiac function parameters in patients with AHF in this study. The possible reason is that the severity of AHF patients included in this study is mild, resulting in less pronounced changes in gastrointestinal structure. Further research is needed.

This study is the first time that a symptom scale has been introduced in the assessment of gastrointestinal function in patients with heart failure. Ultrasound of the gastrointestinal tract can visualize the gastrointestinal function of patients with acute heart failure. Ultrasound monitors gastrointestinal function in real time, prompting clinical adjustment of treatment plan when gastrointestinal function continues to decline. If the gastrointestinal impairment of AHF patients is severe, it will prolong the hospitalization time, increase the incidence of complications during hospitalization, and then affect the prognosis. In addition, if the patient is in a tracheal intubation or coma state, the clinician wants to understand the patient's gastrointestinal function. At this time, gastrointestinal ultrasound can provide valuable and timely clinical information to devise appropriate therapeutic measures.

Limitations of this study: According to the *post hoc* power calculation, assuming an effect size of 0.5 (medium), a statistical power of 0.61 is obtained. This indicates that the results obtained from the current sample size are still insufficient to fully represent the difference between the two groups, and further research, with well-powered samples, is warranted. AHF is a dynamic condition, and GI symptoms may fluctuate with changes in cardiac function and fluid status. It would be valuable to know whether GI parameters change over time in response to heart failure management or disease progression. Longitudinal studies have not been conducted due to the short follow-up time and the inability to achieve regular retesting for most patients. In the future, we will continue to follow up the patients and collect the GI parameters to observe whether the GI parameters change with the management of heart failure or disease progression, in order to help clinicians adjust the treatment plan in a timely manner to improve the prognosis of patients. The results of the chinese GSRS are related to patients' own sensitivity and are subjective to a certain extent, and they have not been applied to the diagnosis of the gastrointestinal tract in patients with heart failure. A large number of studies are needed to prove their performance in measuring gastrointestinal symptoms in patients with heart failure. Larger-scale studies are needed to further understand gastrointestinal changes in AHF and to evaluate these changes before and after AHF therapy to predict treatment efficacy and guide clinical treatment.

## 5 Conclusion

This study found that patients with AHF exhibited thickening of the gastrointestinal wall and generally reduced gastrointestinal motility, with predominantly lower abdominal symptoms. These findings suggest that ultrasound can effectively monitor the gastrointestinal structure and function of patients with AHF in real-time, and timely detect potential issues, which is expected to support clinical diagnosis and allow to devise appropriate therapeutic measures.

## Data Availability

The original contributions presented in the study are included in the article/Supplementary Material, further inquiries can be directed to the corresponding author.
